# The Anti-Pseudomonal Peptide D-BMAP18 Is Active in Cystic Fibrosis Sputum and Displays Anti-Inflammatory In Vitro Activity

**DOI:** 10.3390/microorganisms8091407

**Published:** 2020-09-12

**Authors:** Margherita Degasperi, Chiara Agostinis, Mario Mardirossian, Massimo Maschio, Andrea Taddio, Roberta Bulla, Marco Scocchi

**Affiliations:** 1 Department of Life Sciences, University of Trieste, 34127 Trieste, Italy; margherita.degasperi@phd.units.it (M.D.); rbulla@units.it (R.B.); 2 Institute for Maternal and Child Health, IRCCS Burlo Garofolo, 34134 Trieste, Italy; chiara.agostinis@burlo.trieste.it (C.A.); massimo.maschio@burlo.trieste.it (M.M.);; 3 Department of Medical Sciences, University of Trieste, 34125 Trieste, Italy; mmardirossian@units.it; 4 Department of Medicine, Surgery and Health Sciences, University of Trieste, 34127 Trieste, Italy

**Keywords:** antimicrobial peptide, cystic fibrosis, *P. aeruginosa* infection, inflammation, BMAP-18

## Abstract

Most Cystic Fibrosis (CF) patients succumb to airway inflammation and pulmonary infections due to *Pseudomonas aeruginosa*. *D*-BMAP18, a membrane-permeabilizing antimicrobial peptide composed of D-amino acids, was evaluated as a possible antibacterial aimed to address this issue. The antipseudomonal activity of *D*-BMAP18 was tested in a pathophysiological context. The peptide displayed activity against CF isolates of *Pseudomonas aeruginosa* in the presence of CF sputum when combined with sodium chloride and DNase I. In combination with DNase I, *D*-BMAP18 discouraged the deposition of new biofilm and eradicated preformed biofilms of some *P. aeruginosa* strains. In addition, *D*-BMAP18 down regulated the production of TNF-α, IL1-β, and TGF-β in LPS-stimulated or IFN-γ macrophages derived from THP-1 cells indicating an anti-inflammatory activity. The biocompatibility of *D*-BMAP18 was assessed using four different cell lines, showing that residual cell-specific cytotoxicity at bactericidal concentrations could be abolished by the presence of CF sputum. Overall, this study suggests that *D*-BMAP18 may be an interesting molecule as a starting point to develop a novel therapeutic agent to simultaneously contrast lung infections and inflammation in CF patients.

## 1. Introduction

Lung diseases are the main cause of mortality in cystic fibrosis (CF). Most CF patients succumb to respiratory failure brought on by chronic bacterial infection and airway chronic inflammation [1,2,3,4]. *Pseudomonas aeruginosa* is the major bacterial pathogen and the most harmful because of its evolution to a persistent phenotype [5]. Intensive antibiotic therapy is required to maintain lung function and to eradicate bacterial pathogens, but nowadays the anti-infective treatment has to face the emergence of multidrug-resistant (MDR) pathogen strains [6,7,8]. This problem is worsened by the adaptation of pathogens to the CF pulmonary environment, and by the formation of biofilms [9].

Antimicrobial peptides (AMPs) are a class of molecules that have attracted considerable interest for development as novel drugs to treat multi-drug resistant (MDR) infections [10,11]. AMPs are naturally part of the innate immune system. They have important roles in the host’s defense, showing broad activity spectrum and a slow rate of acquired resistance by pathogens [12].

The therapeutic potential of AMPs in in vitro CF conditions, i.e., presence of DNA or mucine or in 10% diluted sputum has already been reported [13,14,15]. Among AMPs, cathelicidins showed good antimicrobial activity in vitro against the MDR clinical isolate of *P. aeruginosa* and in vivo, in a murine model of pulmonary infection [16,17,18,19]. Recently, *D*-BMAP18, the enantiomeric form of the α-helical BMAP18, was shown to be stable in the murine pulmonary environment, preserving good antimicrobial activity against planktonic CF-related pathogens [20]. However, its activity had not already been assayed under CF conditions, i.e., in the presence of *P. aeruginosa* biofilm or in CF mucus. Moreover, we also tested its activity as an anti-inflammatory compound, acting in suppressing damaging lung hyper-inflammation.

The aim of this work is to investigate the in vitro activity of *D*-BMAP18 in a pathological CF milieu. First of all, the anti-pseudomonal activity of *D*-BMAP18 was evaluated in CF sputum; we successively analyzed its anti-biofilm function against *P. aeruginosa* strains and in order to investigate possible synergisms, we evaluated the antimicrobial activity of *D*-BMAP18 in co-administration with compounds already approved for CF treatment. Finally, the cytotoxicity and anti-inflammatory activity of the peptide were tested on several eukaryotic cell lines.

## 2. Materials and Methods

### 2.1. Peptides and Antibiotics

*D*-BMAP18 (GRFKRFRKKFKKLFKKLS-am) at ≥ 95% purity, purchased from NovoPro Bioscience Inc. (Shangai, China) was resuspended in HCl 10 mM, vacuum-dried three times from HCl 10 mM and finally resuspended in H_2_O. Tobramycin was purchased from Sigma-Aldrich (St. Louis, MO, USA) and resuspended in H_2_O at 5mg/mL final concentration. All the peptides and antibiotics were stored at −20 °C.

### 2.2. Bacterial Strains and Culture Conditions 

*P. aeruginosa* PA03, PA05, PA07, PA08, PA09, PA10, PA14, PA21, PA31, and PA35 strains were obtained from CF patients as previously reported [18]. Each isolate was collected from a single patient and was resistant to at least three of the following groups of antibiotics: β-lactams with or without β-lactamase inhibitor, aminoglycosides, fluoroquinolones, folate-pathway inhibitors (trimethoprim-sulphamethoxazole), tetracyclines, and macrolides. *P. aeruginosa* RP73, and PAO1 were used as reference strains. All strains were stored at −80 °C until use and plated on Mueller–Hinton agar (MHA; Oxoid S.p.A., Milan, Italy).

### 2.3. Killing Activity of D-BMAP18 in Cystic-Fibrosis Sputum

Sputum samples from CF patients were kindly collected by Dr. M. Maschio at the Institute for Maternal and Child Health IRCCS “Burlo Garofolo”, Trieste, Italy. They were pooled and divided in aliquots that were stored at −20 °C. Bacterial killing assay was performed against *P. aeruginosa* strain RP73 in modified SCFM (Synthetic Cystic Fibrosis Sputum Medium) [18,21] or in CF sputum diluted to 25% *v*/*v* in modified SCFM. *D*-BMAP18 or tobramycin were incubated for 4h using a water bath at 37 °C with 300 mM NaCl (to a final tested concentration of NaCl = 450 mM) and/or with a final tested concentration of 128 μg/mL of bovine DNase I (Sigma-Aldrich, St. Louis, MO, USA) in a final volume of 100 μL of SCFM or 25% CF sputum containing 106 CFU/mL. Samples were then diluted 10-fold in SCFM and 25 μL of each dilution was plated on MHA incubated o/n at 37 °C for viable colony count. Dilutions of the CF sputum alone were also plated on MHA to evaluate its contamination by the endogenous microbiota. No growth of endogenous bacteria was observed in the sputum within the indicated time.

### 2.4. Inhibition of Biofilm Deposition and Eradication

Inhibition of biofilm formation was evaluated against the *P. aeruginosa* PAO1, PA03, PA05, PA07, PA08, PA09, PA10, PA14, PA21, PA22, PA31, PA35, and RP73 strains using the MTT assay as previously described by Mardirossian and colleagues [17]. Biofilm eradication assays were performed against *P. aeruginosa* strains PAO1, PA08, PA09, and RP73, as previously reported by Bonaventura et al. [18]. After an 18 h incubation in MH broth in 96-well flat-bottom microtiter plates (Sarstedt, Milan, Italy), the bacterial biofilms were incubated 24 h at 37 °C with different concentrations of *D*-BMAP18 in 50 μL of MH broth. Before peptide addition, some samples were pre-incubated for 1h at 37 °C in the absence or presence of 128 μg/mL of bovine DNase I (Sigma-Aldrich), or 10 mg/mL of N-Acetyl-L-cysteine (Sigma-Aldrich) or 1 mg/mL of Alginate lyase (Sigma-Aldrich). After incubation, the MTT assay was performed as reported [17].

### 2.5. Cytotoxicity Tests Against Human Cell Lines

The A-549 human pulmonary cell line and HaCaT human keratinocyte cell line (ATCC® CCL-185) were grown in Dulbecco’s MEM (Sigma-Aldrich, USA) with 10% FBS (Euroclone, Milan, Italy) and 100 μg/mL penicillin-streptomycin (Pen-Strep, Sigma-Aldrich) with addition of 2.4 mM glutamine (Gln). Human lymphoid MEC-1 cells (DSMZ, ACC 497) and the monocyte cell line THP-1 (ATCC TIB-202) were grown in RPMI 1640 Medium (Sigma-Aldrich, St. Louis, MO, USA) with 10% FBS, 2 mM Gln and 100 μg/mL Pen-Strep.

A total of 20,000 A-549 or HaCaT cells, or 10,000 MEC-1 or THP-1 cells were seeded in a volume of 50 μL in each well of a 96-well flat-bottom microtiter plate (Sarstedt, Milan, Italy) and incubated o/n at 37 °C. Two-fold serial dilutions of *D*-BMP18 were prepared in vials using the same cell growth medium. Next, 50 μL of these *D*-BMP18 solutions were added to each well containing the cells. After a 20 h incubation at 37 °C in 5% CO_2_, cells were incubated with 20 μL of MTT (5 mg/mL). After 4h incubation at 37 °C, 100 μL of 10% (*w*/*v*) igepal (Sigma-Aldrich, St. Louis, MO, USA) in 0.01 N HCl were added to each well and the plates incubated o/n at 37 °C. Cytotoxicity was estimated as the reduction of medium absorbance at 620 nm by using a Nanoquant infinite M200pro (Tecan, Mannendorf, Swiss) microplate reader. Using the HaCat cells the cytotoxic assay was also performed in heat-inactivated 25% CF sputum in cell media. In the heat-inactivation, the sputum was heated at 110 °C for 20 min and the sterility was checked by o/n plating in MHA.

### 2.6. Anti-Inflammatory Property of D-BMP18 on THP-1 Cells

THP-1 cells (600,000/well) were seeded in 1 mL of medium containing 10 μg/mL of Phorbol 12-myristate 13- acetate (PMA, Sigma-Aldrich, St. Louis, MO, USA) in a 24-well flat-bottom plate (Costar, St. Louis, MO, USA) and were incubated for 2 days at 37 °C in 5% CO_2_. Next, the PMA was removed and the cells were incubated o/n with fresh medium. Then the pro-inflammatory stimuli (10 ng/mL Ultrapure lipopolysaccharide LPS from *E. coli* 0111:B4 strain, Sigma-Aldrich USA, or 5000 U/mL IFN-γ, Sigma-Aldrich, USA) and/or the peptide were added to the cells and the plate was incubated for 24 h at 37 °C in 5% CO_2_. After incubation the RNA extraction, cDNA synthesis, and the real-time quantitative PCR (RT-qPCR) were performed as previously reported [22]. The relative amount of gene production in each sample was determined by the Comparative Quantification (CQ) method supplied as part of the Rotor Gene 1.7 software (Corbett Research, Cambridge, UK) [23] normalizing the values with 18S and expressing as arbitrary units (AU) considering 1 AU obtained from resting macrophages as calibrator. Primer sequences are reported in Table 1.

The level of TNF-α in the supernatants collected from the blood macrophages was measured with commercial ELISA kits following the manufacturer’s instructions (Boster Immunoleader Tema Ricerca, Bologna, Italy).

### 2.7. Isolation and Differentiation of Human Peripheral Monocytes into Macrophages and Their In Vitro Polarization

Peripheral blood mononuclear cells (PBMCs) were isolated as described by Rami and colleagues (23) using Ficoll-Paque Plus density gradient (GE Healthcare Euroclone, Milan, Italy). Fully differentiated macrophages were obtained by culturing 10^6^ monocytes/mL for 7 days at 37 °C and 5% CO_2_ with the same medium as described above, replaced twice a week.

### 2.8. Anti-Inflammatory Property of D-BMAP18 on Macrophages Differentiated Cells

After 7 days, monocytes-derived resting macrophages (RM) were incubated with 5 μg/mL or 25 μg/mL of *D*-BMAP18 and LPS (10 ng/mL). After a 30h incubation the supernatants were collected for the ELISA assay and the RT-qPCR was performed [24].

### 2.9. Statistical Analysis

Statistical analysis was performed on samples analyzed at least in triplicate, and experiments repeated three times. Differences between groups were evaluated using the unpaired Student *t* test (biofilm formation), or the ANOVA test (cytotoxicity).

## 3. Results

### 3.1. Antipseudomonal Activity of D-BMAP18 in CF Sputum

**Figure 1:**
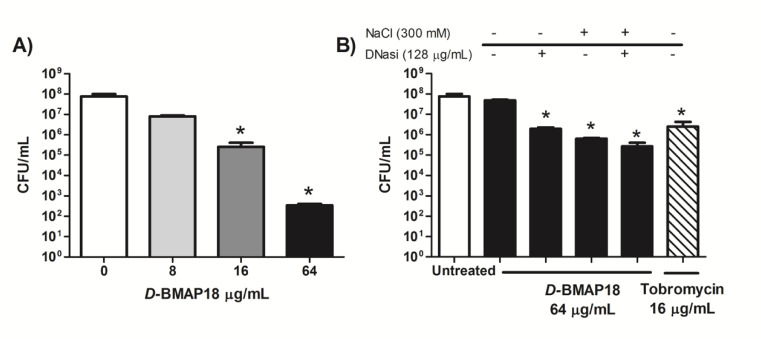
*D*-BMAP18 antimicrobial activity against *P. aeruginosa* RP73. Effect in Synthetic Cystic Fibrosis Sputum Medium (SCFM) medium (**A**) and with the addition of 25% Cystic Fibrosis (CF) sputum in absence or presence of DNase I (**B**). Viable bacterial cells (CFU/mL), have been counted after 4h incubation with *D*-BMAP18 at different concentrations of tobramycin (16 μg/mL) in absence or presence of DNase I (128 μg/mL) and/or NaCl (300 mM). Results are the average of three independent experiments in internal duplicate (*n* = 6). * *p* < 0.05 versus untreated bacteria using the Student *t* test.

The killing activity of *D*-BMAP18 against *P. aeruginosa* was evaluated in SCFM medium and in 25% CF sputum diluted in SCFM medium to assess peptide activity in conditions mimicking the in vivo environment. The peptide incubated for 4 h with *P. aeruginosa* RP73 caused a total breakdown of the bacteria in SCFM (Figure 1A). In contrast, the presence of 25% CF sputum completely inhibited its activity (Figure 1B). Interestingly, the addition in the samples of 300 mM NaCl and/or 128 μg/mL DNase I, both routinely used in therapy for mucus thinning [25], partially rescued the antibacterial activity (Figure 1B). In the presence of both NaCl and DNase I, *D*-BMAP18 reduced the bacterial load by more than 2 logs in CF sputum, a higher effect than that displayed by tobramycin used alone (Figure 1B). These results indicate that *D*-BMAP18 displays antipseudomonal activity in physio pathological conditions resembling the in vivo environment. 

### 3.2. Activity of D-BMAP18 Against Biofilm Deposition 

**Figure 2:**
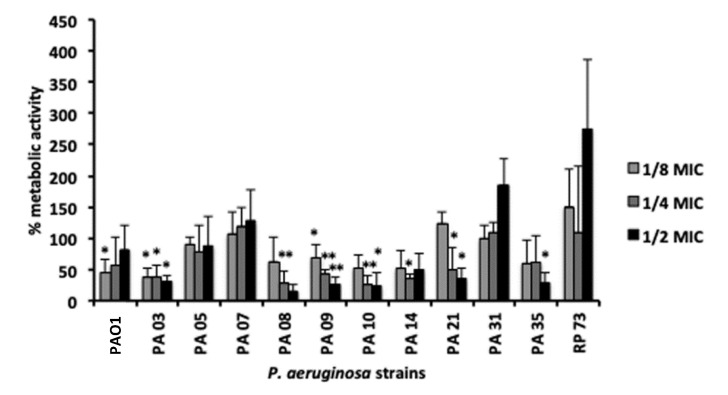
*D*-BMAP18 inhibition of biofilm formation against CF isolates of *P. aeruginosa* strains evaluated by MTT assay. Percentages of metabolic activity of biofilm compared with the untreated controls (100%) are shown. The results are the average of three independent experiments performed as internal triplicate (*n* = 6). * *p* < 0.05, ** *p* < 0.01. Student *t* test, treated *vs* untreated cells.

Previously, we determined the in vitro antimicrobial activity of *D*-BMAP18 against planktonic forms of 10 CF clinical isolates of *P. aeruginosa* [26]. The peptide displayed similar activity, with MIC = 8–16 µg/mL, against all of them. In this study, the effects of *D*-BMAP18 on biofilm deposition have been evaluated against the same panel of the clinical isolates at sub-inhibitory peptide concentrations (from ½ to ⅛ MIC). *D*-BMAP18 inhibited biofilm formation of 8 out 12 strains, in a concentration-dependent manner. Biofilm deposition was significantly inhibited by ½ MIC *D*-BMAP18 of the PAO1, PA08, PA09, PA10, PA21, and PA35 strains (Figure 2) and a reduction of viable cells in the biofilm was still detectable in most strains when the peptide was used at ⅛ MIC.

This effect was strain-specific. In the case of PA07, PA31, and RP73 strains the presence of sub-inhibitory amounts of peptide (from ⅛ to ½MIC values) stimulated the growth of the biofilm. 

### 3.3. Activity of D-BMAP18 Against Preformed Biofilms

**Figure 3:**
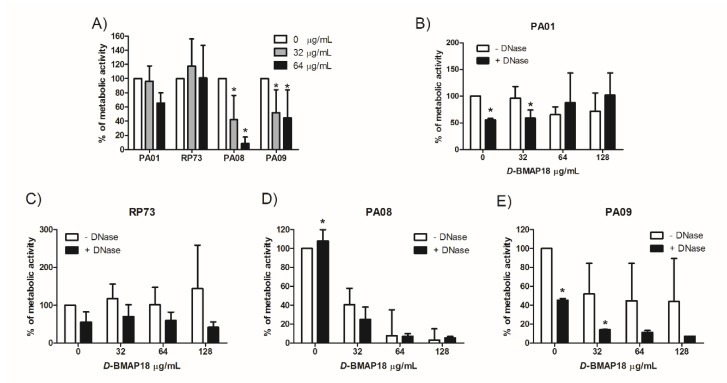
Biofilm eradication activity of *D*-BMAP18. MTT viability test on preformed biofilms has been performed on four *P.a.* strains using the peptide at 4× and 8× MIC values (**A**). Assays have been also performed after 1h pretreatment with (+) or without (-) DNase I (128 μg/mL) on PAO1 (**B**), RP73 (**C**), PA08 (**D**), and PA09 (**E**) strains. Percentage of metabolic active biofilm after 24 h treatment in comparison with the untreated control of growth is shown. The results are the average of three independent experiments in internal triplicate (*n* = 9). * *p* < 0.05. Student *t* test comparing samples with/without DNase.

The activity of *D*-BMAP18 on preformed biofilm was evaluated on *P. aeruginosa* PAO1 and RP73 reference strains, and on the clinical isolates PA08 and PA09 selected for their low variability in the biofilm inhibition assay. Concentration-dependent reduction of biofilm was observed for the PA08 and PA09 strains exposed to 32 μg/mL and 64 μg/mL (4× and 8× MIC) *D*-BMAP18 but not for PAO1 and RP73. The PA09 biofilm was 50% erased and the PA08 biofilm was more than 90% erased at 64 μg/mL. In contrast, RP73 was not susceptible to the antibiofilm activity of the peptide, a result in line with that of the inhibition deposition assay. This assay was performed in parallel using tobramycin as a control; the anti-biofilm activity of the peptide is comparable to that of this antibiotic (Supplementary material Figure S1). Activity on preformed biofilms was also studied after 1h pre-treatment of the bacterial biofilm with DNase I, an enzyme that degrades the eDNA of the extracellular polymeric substance. The presence of the hydrolase enhanced the *D*-BMAP18 eradicating activity against PA08 and PAO1 and especially PA09 biofilms (Figure 3). While the biofilm reduction of the PAO1 strain was likely due to DNase activity alone, in the case of PA09 the combined activity of *D*-BMAP18 and DNase seemed to be very effective with 80% reduction of the biofilm mass at 4 × MIC. Pre-treatments of samples with N-acetylcysteine, a mucolytic agent approved for therapy, or alginate lyase, for degrading the bacterial alginate, did not significantly enhance the activity of the peptide.

### 3.4. In vitro Cytotoxicity of D-BMAP18 Against Human Cell Lines

**Figure 4:**
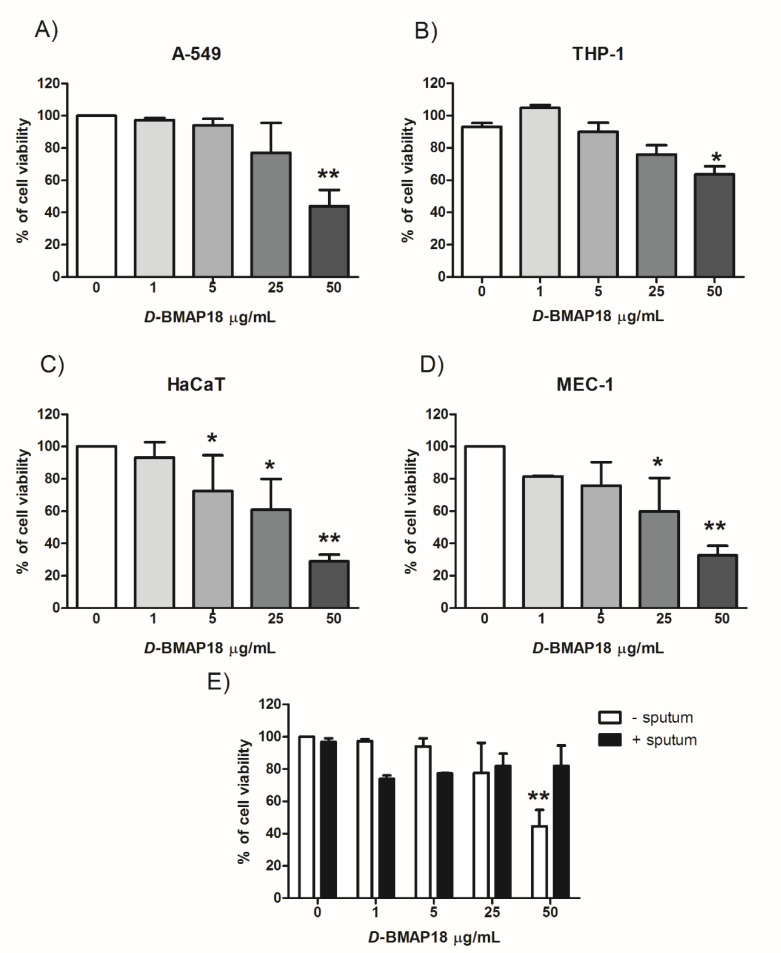
Effects of *D*-BMAP18 on the viability of human cell lines. MTT assay against lung carcinoma A-549 cell line (**A**), monocytic THP-1 cells (**B**), keratinocyte HaCaT cells (**C**), and lymphocytic leukemia MEC-1 cells (**D**) are shown. Data are expressed as percentages of viable cells compared to the untreated control after 24 h of treatment with different concentrations of *D*-BMAP18. (**E**): *D*-BMAP18 cytotoxicity of pulmonary A-549 cell line in the presence and in the absence of 25% CF sputum. The results are the average of three independent experiments in internal triplicate (*n* = 9). * *p* < 0.05, ** *p* < 0.01, Student *t* test, treated vs untreated cells.

We investigated possible adverse effects of *D*-BMAP18 on different human eukaryotic cells. Cytotoxicity assay was performed on the lung carcinoma A-549, immortalized keratinocyte HaCaT, lymphocytic leukemia MEC-1, and leukemia monocytic THP-1 cell lines, by incubating each cell line for 24 h with *D*-BMAP18 up to 50 μg/mL. Viability of THP-1 and A549 was not affected up to 25 μg/mL peptide (Figure 4), while at 50 μg/mL a reduction of 35% and 50%, respectively, was observed. MEC-1 cells started to suffer in the presence of 25 μg/mL *D*-BMAP18, and the HaCat cells were the most susceptible since they were already slightly affected at 5 μg/mL. 

Since we tested the antibacterial activity of *D*-BMAP18 in the presence of interfering substances contained in the CF sputum, we evaluated its possible influence on peptide activity against eukaryotic cells. Cytotoxicity tests on A-549 cells were repeated in the presence of heat inactivated 25% CF sputum. Under these conditions, *D*-BMAP18 did not reduce A-549 viability even at 50 μg/mL, suggesting that the substances present in sputum reduced the adverse effects of the peptide on the cells as well as making it less active towards the bacteria. 

### 3.5. D-BMAP18 Reduces the Bacterial Pro-Inflammatory Stimulus In Vitro

**Figure 5:**
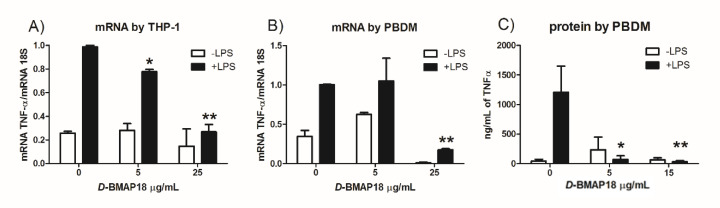
*D*-BMAP18 anti-inflammatory activity on THP-1 cells. (**A**,**B**) RT-qPCR expression analysis of TNF-α in LPS stimulated THP-1 cells treated with *D*-BMP18. After 24 h treatment with 10 ng/mL LPS, and/or *D*-BMAP18 at 5 or 25 μg/mL, total mRNA of THP-1 (**A**) or PBDM (**B**) was isolated and gene expression analysis was performed by RT-qPCR. The expression of TNF-α mRNA was normalized to housekeeping mRNA 18S. The calibrator condition representing the cytokine expression of macrophages after stimulation with only LPS, which we considered our reference value, was 1AU. The results are the average of two independent experiments in internal duplicates (*n* = 4). * *p* < 0.05, ** *p* < 0.01. Student *t*-test evaluated against the untreated cells. (**C**) ELISA assay evaluating the level of the pro-inflammatory cytokine TNF-α after a 36h treatment with LPS and *D*-BMAP18 at 5 and 15 μg/mL. The results are the average of two independent experiments in internal duplicates (*n* = 4). * *p* < 0.05, ** *p* < 0.01. Student *t*-test.

To investigate whether *D*-BMAP18 also displays anti-inflammatory activity, the macrophage-like THP-1 cell line was stimulated with LPS, as the potent pro-inflammatory stimulus, in the presence or absence of different concentrations of *D*-BMAP18. The potential anti-inflammatory activity of the peptide was evaluated on LPS-treated THP-1 cells, measuring the expression level of the cytokine TNF-α, a marker of inflammation. RT-qPCR analysis indicated that *D*-BMAP18 was able to down-regulate TNF-α mRNA expression in a concentration-dependent manner in LPS-stimulated macrophages (Figure 5A). This observation was also confirmed on peripheral blood-derived macrophages (PBDM) isolated from two different donors. In this case the peptide itself, at the lower tested concentrations, caused a weak increase in the expression of the cytokine, but overall it reduced the intense signal obtained by using LPS as stimulus (Figure 5B). We confirmed the effectiveness of *D*-BMAP18 in reducing the expression of TNF-α induced by LPS also titrating by ELISA the level of this cytokine in 36 h cell supernatant. To avoid the possibility that the effect observed in the treatment with a higher concentration of peptide was due to a partial cellular suffering, we used an intermediate peptide concentration (15 µg/mL). As shown in Figure 5C, also in this case, a strong reduction of TNF-α synthesis was observed in LPS-treated PBDM in the presence of *D*-BMAP18 with both concentrations.

### 3.6. The Overall Anti-Inflammatory and Anti-Fibrotic Activity of D-BMAP18

**Figure 6:**
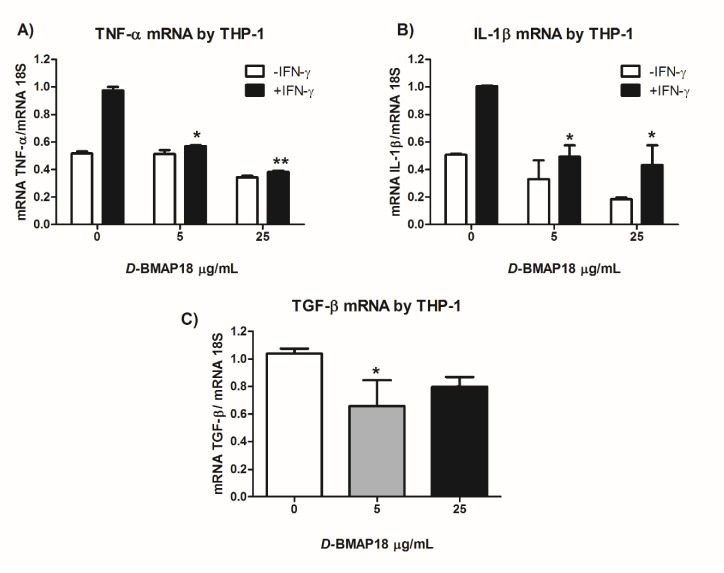
*D*-BMAP18 anti-inflammatory activity on THP-1 cells. RT-qPCR analysis of the pro-inflammatory cytokines (**A**) TNF-α; (**B**) IL1-β and anti-inflammatory cytokine; (**C**) TGF-β after 24 h treatment with IFN-γ (5000 U/mL), and/or D-BMP18 at 5 and 25 μg/mL. The cytokine expression in presence of IFN-γ and *D*-BMAP18 was expressed as fold increase compared to the IFN-γ alone. The results are the average of two independent experiments in internal duplicates (*n* = 4). * *p* < 0.05, ** *p* < 0.01. Student *t*-test.

To better characterize the anti-inflammatory properties of *D*-BMAP18, we analyzed the capability of this peptide to interfere with the production of cytokines by macrophages stimulated with IFN-γ. To this aim, we investigated the macrophage expression of the pro-inflammatory cytokines TNF-α and IL-1β in response to IFN-γ, in the presence or absence of *D*-BMAP18. The results in Figure 6 show that *D*-BMAP18 reduced the expression of TNF-α and IL-1β by IFN-γ-stimulated macrophages.

In parallel to the anti-inflammatory activity, the inhibition of TGF-β expression by *D*-BMAP18, a cytokine well known for its involvement in fibrosis, was investigated. The results showed a great decrease in TGF-β expression by macrophages in the presence of 5µg/mL of peptide, compared to the untreated cells (Figure 6C). 

## 4. Discussion

Novel effective antimicrobial agents are urgently required for the pharmacological therapy of CF patients with pulmonary infections. Previously, we reported that BMAP18, the N-terminal portion of the natural peptide BMAP28, is strongly active *in vitro* against several CF clinical isolates of *P. aeruginosa* and *S. maltophilia* [17] and that its all-D enantiomer *D*-BMAP18 is stable when exposed to mice protease-rich bronchoalveolar fluid [20]. In this study we showed that this defense peptide is also effective in *P. aeruginosa* biofilm formation and eradication and determined the conditions to maintain its activity in media reproducing the physiopathological context of infected lungs. Moreover, we showed that BMAP18 exerts anti-inflammatory activity possibly helping avoid the formation of pulmonary fibrosis [26].

In *P. aeruginosa*, the ability to form biofilm enhances its ability to cause persistent infections and contributes to its ability to colonize and persist in acute and chronic infections by protecting bacteria from host defenses and chemotherapy [27]. We reported here that in vitro the peptide inhibited the formation of new biofilm and eradication of pre-formed biofilm of most *P. aeruginosa* CF isolates tested, comparably to tobramycin. This feature has been described for other AMPs. The antibiofilm activity of *D*-BMAP18 is in line with or even better than other AMPs considering its small size (2343 kDa) [28]. An example is LL-37 which reduced the formation of *P. aeruginosa* biofilm in a similar manner but which, unlike *D*-BMAP18, is unstable and shows higher cytotoxicity [29,30]. For some bacterial strains, sub-inhibitory concentrations of the peptide seem to induce biofilm formation. This observation has already been reported for macrolides which alter the formation of the biofilm matrix [31] or for imipenem which induces the production of alginate and peptidoglycan [32]. 

The eDNA is a major component of *P. aeruginosa* biofilm. It has been demonstrated recently that eDNA inhibited positively-charged antimicrobials such as aminoglycosides and antimicrobial peptides. Interestingly, we observed that antibiofilm activity of *D*-BMAP18 is enhanced by the presence of DNase I. The nuclease decreased per se the deposition on new biofilm in 3 out of 4 strains tested, but an additive effect was detected when it was used in combination with *D*-BMAP18. This result could be due to the degradation of eDNA by DNase, thus weakening the biofilm structure and allowing the action of the peptide. Further studies will be necessary to confirm this mechanism.

*D*-BMAP18 lost most of its antipseudomonal activity when assayed in CF sputum, used to mimic the composition of the airway environment. These observations are not surprising given that CF sputum is a complex biological fluid. Tobramycin, one of the drugs commonly used in CF therapy [33], also partially decreased its potency in CF sputum (see Figure 1). Since *D*-BMAP18 is stable to protease degradation, it might be that antibacterial activity is inhibited by unspecific binding with sputum components. Some proteins and lipoproteins contained in bronchoalveolar lavage have been reported to inhibit the activity of cationic molecules [34]. Free DNA has also been abundantly found in the airway fluids of CF patients [35]. The antibacterial activity of *D*-BMAP18 was partially restored by the addition of both sodium chloride (300 mM) and DNase I, and they were most effective in restoring BMAP18 activity when used together. This result suggests that polyanionic molecules such as eDNA contained in CF sputum could electrostatically interact with cationic *D*-BMAP18 and inhibit its activity. The increase of salt concentration likely reduces the electrostatic interactions between the peptide and sputum components while DNase I could degrade the negatively charged eDNA released in lung [36]. Moreover, the peptide has been shown to be highly active in the presence of 450 mM NaCl (300 mM salt added to a medium displaying an ionic strength corresponding to that of 150 mM NaCl). This salt concentration normally suppresses the antibacterial activity of most AMPs and not only that of the salt-sensitive peptides β- defensins [37] and LL-37 [38]. This uncommon and remarkable feature of *D*-BMAP18 combined with the enhanced antibacterial activity observed in the presence of DNase, suggests a potential use of D-BMAP18 in combination with hypertonic solutions and/or dornase alfa (recombinant DNase I), which have already been approved for the mucolytic therapy of CF patients [39]. 

We reported first clues on the anti-inflammatory activity of *D*-BMAP18. *D*-BMAP18 led to a significant reduction of the expression and protein production of TNF-α and IL1-β cytokines in LPS-stimulated THP-1-derived macrophages and in monocytes isolated from blood. This result is in agreement with those of other AMPs, such as the chensinin-1 and temporin-derived peptides, that reduce the expression of pro-inflammatory cytokines such as TNF-α [40,41]. Moreover, the peptide was able to downregulate the expression of TGF-β in macrophages derived by THP-1 cells. This may be a relevant result, if confirmed, because of the important role of TGF-β in inducing pulmonary fibrosis linked with CF [42], and because the state of hyper-inflammation is one of the major causes of mortality in CF patients [1]. Nowadays, most CF therapies focus on treating secondary pulmonary complications, and the importance of targeting TGF-β as a cytokine involved in fibrosis, inflammation, and injury response has already been reported in the literature [43]. For example, the monoclonal antibody STX-100X inhibits TGF-β activation at the site of injury and prevents the development of pulmonary fibrosis [44]. 

A major concern about the use of HDPs as a therapeutic agent arises from their non-negligible cytotoxicity mainly due to low selectivity against the prokaryotic membranes [45]. We tested the effects of *D*-BMAP18 at high concentrations against four different human cell lines. We found that eukaryotic cell lines are differently susceptible in vitro to the peptide. THP-1 and A549 appeared to be more resistant to the treatment, displaying no adverse effect up to 25–50 μg/mL, while MEC-1 and HaCat cells were more susceptible. Similar results have been obtained in a previous study. This result is in agreement with those observed for other antimicrobial peptides such as temporin A and protegrin [46]. The different susceptibility among different cell lines could be due to differences in membrane composition, a hypothesis also reported for the lytic antimicrobial peptide Bovicin HC5, which was more toxic against the Vero epithelial cells than against the breast MCF-7 cells [47]. It has already been reported that different amounts of cholesterol among eukaryotic cell membranes enhances the rigidity of lipid bilayers, reducing or inhibiting membrane disruption by antimicrobial peptides [48].

It is worth noting that, at these concentrations (16–64 µM), *D*-BMAP18 decreased 2–4 logs of the *P. aeruginosa* cells in SCFM medium after only 4h incubation. The high cell selectivity of the peptide can be appreciated more on the basis of cytotoxicity tests performed in the presence of 25% CF sputum, added in the samples to approach the conditions used for testing antipseudomonal activity. In these conditions A549 cells are fully protected against any damaging effects of *D*-BMAP18 even at 50 μg/mL while it continued to kill *P. aeruginosa* cells. This aspect needs to be further clarified in the future since it is linked to the possibility of widening the therapeutic scope of this molecule. 

## 5. Conclusions

In conclusion this study suggests that *D*-BMAP18 might be effective in the *in vivo* context through co-administration with mucolytic agents, also being active against the sessile form of *P. aeruginosa*. Moreover, its anti-inflammatory effects, if confirmed, could contribute to beneficial treatment of CF lung infections. *D*-BMAP18 seems to need optimization to further reduce detectable cytotoxicity at least against susceptible cells. To overcome this drawback, derivatives of *D*-BMAP18 as pro-drugs are in preparation.

## Figures and Tables

**Table 1:** Primer used for RT-qPCR analysis.

Gene	Tm	Sense	Sequence	Accession Number
*18S*	60	Forward	ATCCCTGAAAAGTTCCAGCA	NM_022551.2
		Reverse	CCCTCTTGGTGAGGTCAATG	
*TNF-α*	66	Forward	GGCCCAGGCAGTCAGATCAT	NM_000594.3
		Reverse	GGGGCTCTTGATGGCAGAGA	
*IL-1β*	60	Forward	GTACATCCTCGACGGCATC	NM_000600.3
		Reverse	CCAGGCAAGTCTCCTCATTG	

Abbreviations: 18S ribosomal RNA (18S), interleukin (IL), tumor necrosis factor-α (TNF-α), melting temperature (Tm).

## Supplementary Materials

These are available online at https://www.mdpi.com/2076-2607/8/9/1407/s1.
