# Supplementary material for: The Anti-Pseudomonal Peptide D-BMAP18 Is Active in Cystic Fibrosis Sputum and Displays Anti-Inflammatory In Vitro Activity
Source: Microorganisms. 2020 Sep 12;8(9):1407. doi: 10.3390/microorganisms8091407 (PMC7565916; doi:10.3390/microorganisms8091407)
Supplement: Supplementary file 1 [file microorganisms-08-01407-s001.pdf]

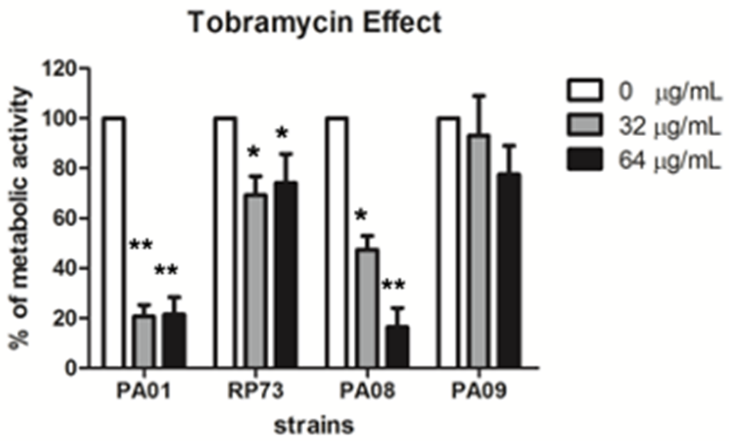

Figure S1. Biofilm eradication activity of Tobramycin. MTT viability test on preformed biofilms on PAO1, RP73, PA08 and PA09. Percentage of metabolic active biofilm after 24h-treatment in comparison with the untreated control of growth is shown. The results are the average of three independent experiments in internal triplicate ($n = 9$). **$p < 0.01$, *$p < 0.05$. Test t-student comparing samples with/without treatment.
